# Efficacy of an asynchronous electronic curriculum in emergency medicine education in the United States

**DOI:** 10.3352/jeehp.2017.14.29

**Published:** 2017-12-11

**Authors:** Alisa Wray, Kathryn Bennett, Megan Boysen-Osborn, Warren Wiechmann, Shannon Toohey

**Affiliations:** 1 Department of Emergency Medicine, Irvine School of Medicine, University of California, Orange, CA, USA; 2 Irvine School of Medicine, University of California, Irvine, CA, USA; Hallym University, Korea

**Keywords:** Curriculum, Emergency medicine, Graduate medical education, Retrospective studies, United States

## Abstract

**Purpose:**

The aim of this study was to measure the effect of an iPad-based asynchronous curriculum on emergency medicine resident performance on the in-training exam (ITE). We hypothesized that the implementation of an asynchronous curriculum (replacing 1 hour of weekly didactic time) would result in non-inferior ITE scores compared to the historical scores of residents who had participated in the traditional 5-hour weekly didactic curriculum.

**Methods:**

The study was a retrospective, non-inferiority study. conducted at the University of California, Irvine Emergency Medicine Residency Program. We compared ITE scores from 2012 and 2013, when there were 5 weekly hours of didactic content, with scores from 2014 and 2015, when 1 hour of conference was replaced with asynchro-nous content. Examination results were compared using a non-inferiority data analysis with a 10% margin of difference.

**Results:**

Using a non-inferiority test with a 95% confidence interval, there was no difference between the 2 groups (before and after implementation of asynchronous learning), as the confidence interval for the change of the ITE was −3.5 to 2.3 points, whereas the 10% non-inferiority margin was 7.8 points.

**Conclusion:**

Replacing 1 hour of didactic conference with asynchronous learning showed no negative impact on resident ITE scores.

## Introduction

Over the past 20 years, asynchronous learning has gained popularity in health professions education. Technology, rather than educational theory, has driven much of this change [1], as advances in technology have allowed for improved venues for communication and greater access to learning materials (e.g., free open access medical education [FOAM], massive open online courses, podcasts/vodcasts, and online textbooks). Asynchronous learning may offer advantages for the current generation of “millennial learners” [2], as it provides a digital learning experience, may encourage a habit of lifelong learning [1], and may be superior or equivalent to formalized didactic lectures [3].

**Fig. 1.**
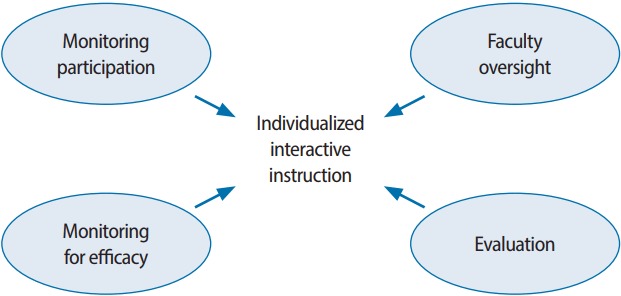
Individualized interactive instruction, as defined by the Accreditation Council for Graduate Medical Education. From Accreditation Council for Graduate Medical Education. Frequently asked questions: emergency medicine [Internet]. Chicago (IL): Accreditation Council for Graduate Medical Education; 2017 [cited 2017 Oct 23]. Available from: https://www.acgme.org/Portals/0/PDFs/FAQ/110_emergency_medicine_ FAQs_2017-07-01.pdf [8].

Self-directed learning (SDL), popularized by Knowles, offers some theoretical background supporting asynchronous learning [4]; however, many have challenged SDL, arguing that learners cannot create their own learning goals [5] and cannot self-assess [6]. Therefore, asynchronous or self-guided learning should be supervised [1]. The Accreditation Council for Graduate Medical Education (ACGME) Program Requirements for Emergency Medicine (EM) allow for up to 20% (1 hour per week) of planned educational content to be supervised asynchronous learning, or “individualized interactive instruction” [7]. Individualized interactive instruction must be overseen by faculty, with the program director monitoring resident participation; it must also be evaluated and monitored for efficacy (Fig. 1) [8].

Graduate medical education is rapidly evolving as the required material to cover grows [9], despite increasing constraints on instructional time and funding. This incongruence may be addressed by asynchronous, individualized, computer-based instruction, which offers a compelling alternative to traditional live didactic instruction. As millennials, today’s medical graduates possess advanced information technology skills and experience in computer-based learning; they are generally comfortable with utilizing technology as a key component of their professional learning [2].

While the significant investment required to design, implement, and evaluate the development of asynchronous content must be considered, the long-term gains in instructor and scheduling time may outweigh these upfront costs. Since millennial learners are naturally drawn to FOAM and other online resources [2], individualized interactive instruction allows program directors and faculty to ensure that the content they access is high-quality and reliable. Other positive externalities include possible increased resident satisfaction via the opportunity to learn at a time and setting convenient and most beneficial for learners.

In 2013, the University of California, Irvine Emergency Medicine Residency Program implemented an iPad-based asynchronous learning curriculum. The objective of this asynchronous curriculum was to teach EM residency core content by replacing 1 hour of the usual residency conference curriculum with asynchronous learning. We sought to use the weekly asynchronous content to attain the same system-based educational objectives as usual conference lectures, while shortening conference (didactic) time by 1 hour. We hypothesized that the implementation of the asynchronous curriculum with reduced in-person didactic time would result in non-inferior resident in-training exam (ITE) scores compared to those of residents who had experienced the traditional 5-hour lecture-based didactic curriculum.

## Methods

The University of California, Irvine Emergency Medicine Residency Program implemented a new asynchronous curriculum in 2013. We decreased the standard lecture time by 20%, from 5 hours per week to 4 hours per week, substituting 1 conference curriculum hour with an asynchronous learning module. The asynchronous curriculum included 4 modules per month, each designed to take 1 hour or less to complete. All modules were available through a Learning Management System (LMS), iTunesU, or Schoology. An iTunesU application on iPads was used during the first year; while in the second year, we used Schoology. We selected Schoology as the continuing LMS because, in our opinion, it provides a more organized platform for our educators and learners. Furthermore, it includes quiz and discussion options that allow learners to receive real-time feedback, enables interaction between residents and faculty, and provides hard stops in the form of module exams that give immediate feedback and require the learner to demonstrate proficiency in one topic before moving onto the next step. The platform additionally allowed the program directors to continuously monitor the residents’ progress.

The monthly modules corresponded to our system-based curriculum (cardiac topics during cardiac block, etc.) and included journal and review articles, audio and video lectures, and podcasts, as well as links to FOAM educational content. All content that was chosen was considered core content that would be appropriate for didactics during conference and would be seen on the ITE. A chief resident with an interest in simulation and technology or a faculty member chose the content for the monthly modules and the program director approved the content. Each module was followed by a short quiz, which was used to monitor completion of the modules and the efficacy of the program. Additionally, 1 module in each block contained a 20-question board-review-style quiz with immediate feedback with in-depth answers and explanations for each question in a format similar to the board-review-style lectures that were previously given during conference. The LMS could be accessed remotely, allowing residents autonomy to consume the modules on their own time and in the location of their choice. The length of time allotted to the asynchronous, web-based modules was self-directed and unlimited, but was not designed to take more than 1 hour for 1 pass. We required documented completion of each module in order to receive credit. We evaluated the residency curriculum yearly during the annual program evaluation.

The study was a retrospective, non-inferiority study. We analyzed ITE scores from 2012 and 2013 when there were 5 hours of conference per week (36 individual resident test scores), as well as from 2014 and 2015 after the asynchronous curriculum component had been implemented (39 individual resident exam scores). The primary outcome was ITE scores. Average United States Medical Licensing Examination (USMLE) step 2 scores were analyzed to ensure there were no differences in baseline test-taking characteristics between classes.

The data were analyzed using Stata ver. 14.0 (Stata Corp., College Station, TX, USA). We used the 2-sample t-test, with a 2-tailed alpha of 0.05, to assess differences between between ITE scores from 2012–2013 (36 residents: 21 males, 15 females) and 2014–2015 (39 residents: 19 male and 20 female). The confidence intervals for differences in proportions were calculated. We set statistical significance at P<0.05. The study was approved by the IRB of University of California, Irvine after receiving informed consent from the subjects (IRB Number: HS# 2013-9899).

## Results

We received consent from a total of 27 residents spanning postgraduate year (PGY)-1 to PGY-3 to participate in the study during the academic years ending in 2012 through 2015. Raw data are available from Supplement 1. We compared 2 score cohorts: (1) ITE scores of residents after implementation of the asynchronous curriculum (4 hours of didactic curriculum plus 1 hour of individualized interactive instruction) and (2) ITE scores of residents before implementation of the asynchronous curriculum (5 hours of in-person didactic curriculum). The new curriculum score cohort included 39 individual ITE exam scores from years 2014 (6 PGY-3s, 6 PGY-2s, and 7 PGY-1s) and 2015 (5 PGY-3s, 7 PGY-2s, and 8 PGY-1s). The traditional curriculum score cohort included 36 individual ITE scores from 2012 (6 PGY-3s, 6 PGY-2s, and 6 PGY-1s) and 2013 (6-PGY-3s, 6-PGY-2s, and 6-PGY-1s). Of note, there were more individual exam scores than individual residents, since residents took the ITE annually, between 1 and 3 times during the study period. To ensure there were no differences in baseline test-taking characteristics between classes, we analyzed the average entering USMLE step 2 scores for each group (Table 1). To account for rising USLME step 2 minimum passing score means, we used the difference between subjects’ scores and the minimum passing score mean, averaged these differences, and compared them using 1-way between-subjects analysis of variance, which showed no statistically significant differences among the classes (F[5,33]= 0.53, P= 0.75).

The mean ITE scores were 77.64± 6.38 for traditional lecture/didactic instruction and 77.08± 6.22 for lecture/didactic plus asynchronous instruction. The 2-tailed non-inferiority t-test revealed a statistically insignificant mean difference between the didactic and asynchronous modalities of 0.56 points (95% confidence interval [CI], −3.46 to 2.34 points; P = 0.7005). These results demonstrate no difference between the 2 groups (before and after implementation of asynchronous learning) given that the CI fell within the 10% non-inferiority margin of 7.8 points (Table 2).

Despite the decrease in conference time from 5 to 4 hours per week, replacing the hour with asynchronous learning showed no negative impact on resident ITE scores.

## Discussion

The results of this study suggest that decreasing the didactic conference time by 20% and replacing it with asynchronous learning did not negatively impact resident ITE scores. Therefore, the incorporation of individualized interactive instruction is feasible and non-inferior to standard lectures. These results support the ACGME decision to incorporate asynchronous, or individualized interactive instruction, learning modalities in the educational program guidelines as of 2013 [7].

Our results align with much of the current literature finding that asynchronous and didactic education are equivalent with regard to knowledge gain outcomes [3,10,11]. There are limited data on this comparison specifically in the EM core curriculum, and some studies have reported negative results [12]; however, other studies on EM procedure instruction through web-based asynchronous platforms have garnered positive findings [10,13].

This study has some limitations that need to be considered when interpreting our findings. Our study was conducted at a single academic EM residency site and had a small sample of subjects. Furthermore, structured didactic curriculum is one of many factors that impact ITE scores. In a multi-center survey of resident study preferences, Knapp et al. [14] identified many preferred study methods that may not be represented by standard didactic sessions, with computer-based learning using question banks regarded as the most highly efficacious by residents. Additionally, Cheng et al. [15] showed that surgical residents who completed more practice questions performed better on ITEs. In an associated vein, Gene Hern et al. [16] showed that greater didactic session attendance did not correlate with an individual’s ITE scores, notwithstanding the presumed pedagogical benefits of traditional lectures in overall learning. Finally, at the time of the incorporation of our asynchronous curriculum, no LMS was able to record and provide the amount of time that users spent on each module; thus, the quizzes were used as a surrogate marker and questions were written to assess whether the modules had been completed.

Inevitably, students studying independently are less likely to receive interactive feedback as readily as is possible in live didactic sessions. There may be more meaningful interactions between teachers and residents during live lectures, enabling the immediate clarification of concepts. Our asynchronous platform did allow for optional discussions between residents and faculty regarding the content, but a more robust interactive component with mandatory discussion boards may increase engagement both among residents and between residents and faculty during individualized interactive instruction activities. This is a potential area to explore if more interactive content is demonstrated to lead to better quiz scores, and is an issue we intend to analyze further.

In conclusion, individualized interactive instruction can be implemented in any residency program and creates an opportunity to teach residents via non-standard means, to direct residents toward high-quality educational resources, and to help residents find resources for lifelong learning. While it can never replace skills-based instructional education or the interaction and immediate feedback fostered by live interactions, web-based asynchronous learning is an exciting complement to traditional education. Combining interactive didactic conferences with asynchronous modalities can capitalize on the respective strengths of both techniques. Ideally, any implemented curriculum should demonstrate at least equivalency with current teaching methods to be adopted long-term. Furthermore, if multiple EM residencies collaborate to develop a standard asynchronous curriculum, a library of content could be built to be shared among residency programs, which would be highly beneficial throughout the medical health education system.

## Figures and Tables

**Table 1.** USMLE step 2 scores

Class	USMLE step 2 scores		
	Average score	USMLE step 2 minimum passing/maximum score	Difference between class average and reported minimum passing score
2012	235	184/270	51
2013	232.83	184/270	48.83
2014	239.3	189/270	50.3
2015	247.17	189/270	58.17
2016	245.67	196/270	49.67
2017	251.83	203/270	48.83

USMLE, United States Medical Licensing Examination.

**Table 2.** Mean test scores

Course format (cohort years)	N	Mean ± standard error	95% confidence interval	P-value
Didactic only (2012 and 2013)	36	77.64 ± 1.06	-2.15 to 2.16	
Didactic+asynchronous (2014 and 2015)	39	77.08 ± 1.00	-4.35 to 7.81	
Difference		0.56 ± 1.46	-3.46 to 2.34	0.7005

## Supplementary materials

Supplement 1. Data file is available from https://doi.org/10.7910/DVN/GEIBIA.

Supplement 2. Audio recording of the abstract.
